# Association of dynamic susceptibility magnetic resonance imaging at initial tumor diagnosis with the prognosis of different molecular glioma subtypes

**DOI:** 10.1007/s10072-020-04474-7

**Published:** 2020-05-28

**Authors:** Cornelia Brendle, Uwe Klose, Johann-Martin Hempel, Jens Schittenhelm, Marco Skardelly, Ghazaleh Tabatabai, Ulrike Ernemann, Benjamin Bender

**Affiliations:** 1 Diagnostic and Interventional Neuroradiology, Department of Radiology, Eberhard Karls University, Hoppe-Seyler-Straße 3, 72076 Tuebingen, Germany; 2 Neuropathology, Department of Pathology and Neuropathology, Eberhard Karls University, Calwerstr. 3, 72076 Tuebingen, Germany; 3 University Hospital for Neurosurgery, Eberhard Karls University, Hoppe-Seyler-Straße 3, 72076 Tuebingen, Germany; 4 Interdisciplinary Section of Neurooncology, Eberhard Karls University, Hoppe-Seyler-Straße 3, 72076 Tuebingen, Germany

**Keywords:** Glioma, DSC-MRI, Perfusion, prognosis, IDH mutation, Molecular

## Abstract

**Purpose:**

The updated 2016 CNS World Health Organization classification differentiates three main groups of diffuse glioma according to their molecular characteristics: astrocytic tumors with and without isocitrate dehydrogenase (IDH) mutation and 1p/19q co-deleted oligodendrogliomas. The present study aimed to determine whether dynamic susceptibility contrast magnetic resonance imaging (DSC-MRI) is an independent prognostic marker within the molecular subgroups of diffuse glioma.

**Methods:**

Fifty-six patients with treatment-naive gliomas and advanced preoperative MRI examination were assessed retrospectively. The mean and maximal normalized cerebral blood volume values from DSC-MRI within the tumors were measured. Optimal cutoff values for the 1-year progression-free survival (PFS) were defined, and Kaplan-Meier analyses were performed separately for the three glioma subgroups.

**Results:**

IDH wild-type astrocytic tumors had a higher mean and maximal perfusion than IDH-mutant astrocytic tumors and oligodendrogliomas. Patients with IDH wild-type astrocytic tumors and a low mean or maximal perfusion had a significantly shorter PFS than patients of the same group with high perfusion (*p* = 0.0159/0.0112). Furthermore, they had a significantly higher risk for early progression (hazard ratio = 5.6/5.1). This finding was independent of the methylation status of O6-methylguanin-DNA-methyltransferase and variations of the therapy. Within the groups of IDH-mutant astrocytic tumors and oligodendrogliomas, the PFS of low and highly perfused tumors did not differ.

**Conclusion:**

High perfusion upon initial diagnosis is not compellingly associated with worse short-term prognosis within the different molecular subgroups of diffuse glioma. Particularly, the overall highly perfused group of IDH wild-type astrocytic tumors contains tumors with low perfusion but unfavorable prognosis.

## Introduction

The 2016 update of the World Health Organization classification of brain tumors (2016 CNS WHO) now combines specific molecular characteristics of gliomas beyond histological characteristics for assignment to different subgroups in an integrated approach. Consequently, diffuse gliomas are classified into three main groups. The first two are astrocytic tumors, which are divided into tumors with isocitrate dehydrogenase (IDH) mutation and without IDH mutation (IDH wild type). The third group, oligodendrogliomas, is defined by IDH mutation, codeletion of chromosomes 1p and 19q, and retention of the ATRX gene [1].

Magnetic resonance imaging (MRI) is the modality of choice for diagnosing brain tumors, as well as assessing the tumor characteristics, the extent at the initial diagnosis, and the therapy response in the course of the disease. Standard MRI is limited to visualizing morphological changes and the disruption of the blood-brain barrier after injection of contrast agent. Beyond that, advanced MRI techniques give insights into the functional properties and tissue composition of gliomas. MR spectroscopy measures biochemical metabolites and can provide direct proof of IDH mutations in gliomas [2].

Diffusion-weighted imaging displays the variable cellularity and tissue structure in different tumor entities and the grade of malignancy [3–5]. Perfusion MRI techniques visualize the vascularization by quantifying the blood flow through the tissue, which is increased in malignant tissue due to neo-angiogenesis [6]. Dynamic susceptibility contrast (DSC)-MRI is the most common perfusion MRI technique. The contrast agent inflow in the T2*-weighted images causes susceptibility-induced signal changes, which are used for calculating the cerebral blood volume (CBV). The absolute perfusion values of DSC-MRI are prone to multiple sources of error, so they are usually corrected using an internal reference, resulting in relative values (rCBV).

DSC-MRI is widely used in the diagnosis of gliomas and has the potential to predict the prognosis of glioma subtypes according to the 2007 WHO classification, particularly in high-grade astrocytomas [7–9]. Previous studies reported that lower tumor perfusion is associated with prolonged survival [7, 8, 10, 11]. DSC-MRI can also help to identify the molecular subgroups of diffuse glioma according to the updated 2016 WHO classification, which differ substantially in their prognosis [12, 13]. However, the genetic profile of a tumor is usually confirmed histologically, and there is only limited knowledge about the prognostic value of DSC-MRI as an independent prognostic factor within a specific glioma group [14].

Therefore, the present study aims to evaluate the 1-year progression-free survival (PFS) of treatment-naïve patients with IDH wild-type astrocytic tumors, IDH-mutant astrocytic tumors, and oligodendrogliomas according to the DSC-MRI perfusion values.

## Materials and methods

### Patients

We retrospectively evaluated all patients with an initial diagnosis of diffuse glioma and clinically indicated advanced MR examination between 11/2012 and 9/2016. All patients provided informed written consent for the scientific use of their data. The local ethics committee approved the study, which was performed in accordance with the Declaration of Helsinki.

The inclusion criteria comprised adult patient age, initial tumor diagnosis, advanced MRI with DSC perfusion before any treatment, pathologic examination including molecular profiling, and follow-up by clinical examination and imaging. We assessed the age, sex, extent of tumor resection (complete versus incomplete), and the application of adjuvant therapy. We gathered follow-up data until progressive disease occurred according to the Response Assessment in Neuro-Oncology (RANO) criteria or until the patients were lost to follow-up [1].

We calculated the PFS from the date of the advanced MR examination to the date of tumor progression. We chose tumor progression and not overall survival as the endpoint because patients in specific glioma groups partially survive for a long time, and the progression-free interval is crucial for quality of life. Furthermore, the overall survival of patients is influenced by variable therapy management in the case of progression.

The IDH mutation status of the tumors was identified using an IDH1 R132H antibody, and rare IDH1/2 mutations in IDH1 R132H-negative cases were identified by pyrosequencing [15, 16]. Nuclear ATRX status was assessed using immunohistochemistry and 1p/19q codeletion by a synthetic high-resolution microsatellite PCR gel [17, 18]. Possible methylation of the O6-methylguanin-DNA-methyltransferase (MGMT) promoter of IDH wild-type astrocytic tumors was identified using methylation-specific pyrosequencing [19]. For further analyses, we differentiated three glioma subgroups according to their molecular characteristics: IDH wild-type astrocytic tumors, IDH-mutant astrocytic tumors (ATRX loss), and oligodendrogliomas (IDH mutation, 1p19q codeletion, ATRX retention).

### MRI examination

All patients were examined on a 3-T MRI scanner (Biograph mMR MR-PET, Siemens Healthcare, Erlangen, Germany) using a head-neck coil. The MR sequences comprised an axial T2-weighted fluid-attenuated inversion recovery (FLAIR) sequence as an anatomical reference (repetition time 9000 ms, echo time 94 ms, inversion time 2500 ms, slice thickness 3 mm, matrix size 207 × 320, field of view 189 × 220 mm^2^). For DSC-MRI, a pre-bolus of contrast agent was applied (0.025 mmol/kg gadobutrol (Gadovist, Bayer Healthcare, Leverkusen, Germany)). Subsequently, a single-shot T2*-weighted echo-planar imaging sequence was performed during the first pass of a contrast agent bolus of 0.1 mmol/kg of gadobutrol (Gadovist, Bayer Healthcare, Leverkusen, Germany; injection rate 3 ml/s). The repetition time was 1130 ms, the echo time was 31 ms, the flip angle was 60°, the slice thickness was 4 mm, the matrix size was 128 × 128, and the field of view was 230 × 230 mm^2^.

### Image analysis

We calculated CBV parametric maps using raw data from DSC-MRI with the software Syngo® MR Perfusion (Siemens Healthineers, Erlangen, Germany) with model-based leakage correction. We manually identified the middle cerebral artery or anterior cerebral artery for the arterial input function. The matrix of the FLAIR sequence was adjusted to the perfusion maps using an in-house Matlab-based application (Matlab 2014b, MathWorks Natick, Massachusetts, USA). We drew a volume of interest (VOI) covering the whole tumor with signal alteration in the FLAIR sequence and excluded surrounding edema, vessels, and necrotic areas [20, 21].

For the reference VOI, we chose a slice in contralateral normal-appearing white matter. We determined the tumor volume in the T2-weighted FLAIR sequence because some tumors did not show contrast enhancement. The VOIs were transferred to the CBV maps automatically. We noted the mean and maximal CBV inside the tumor VOI and the mean values inside the reference VOI. For further analyses, we calculated the relative CBV (rCBV) as the ratio of the perfusion values inside the tumor to values in the reference region.

### Statistical analysis

We performed Spearman’s correlation for the association of 1-year PFS with age and rCBV. We excluded patients who were lost to follow-up within the first year from these analyses. We defined the best rCBV cutoff values for predicting PFS in each separate glioma group by a receiver operating characteristic (ROC) analysis. We performed a Kaplan-Meier survival analysis with a log-rank test for rCBV values, molecular pathological tumor groups, sex, the extent of resection, WHO grades, and MGMT methylation status. We calculated the hazard ratios for the perfusion values. Using the Wilcoxon test, we estimated differences in rCBV between tumor groups and MGMT promoter statuses and set the significance level at *α* = 0.05. We used the software JMP (JMP 11.2.0, SAS Institute, Cary, NC, USA) for the statistical calculations.

## Results

### Patients

Fifty-six patients were included in the present study (mean age 48 ± 16 years, 33 males, 23 females). The median follow-up time was 350 days (range 11–1380 days), so we used the 1-year PFS for further analyses. Tumor progression occurred in 31% of IDH-mutant astrocytic tumors, 46% of IDH wild-type astrocytic tumors, and 13% of oligodendrogliomas. The duration of PFS tended to be higher in cases of IDH-mutant astrocytic tumors than IDH wild-type astrocytic tumors. Oligodendrogliomas had the best prognosis, but the differences between the glioma groups were not significant (*p* = 0.09).

Table 1 shows detailed characteristics of the glioma subgroups. Within the groups, PFS did not correlate with age (*p* = 0.15, *p* = 0.30, and *p* = 0.65, respectively). PFS did not differ between sexes (*p* = 0.46, *p* = 0.90, and *p* = 0.14, respectively) or different extents of resection (*p* = 0.15, *p* = 0.20, and *p* = 0.71, respectively). We could not assess the MR perfusion data in five patients due to incomplete coverage of the tumor by imaging or a lack of arterial input function by insufficient inflow of the contrast agent. Two of these patients had IDH-mutant astrocytic tumors, and three had IDH wild-type astrocytic tumors.

**Table 1:** Patient and tumor characteristics

Parameter	IDH-mutant astrocytoma	IDH wild-type astrocytoma	Oligodendroglioma
Total number	16	24	16
Progress	5	11	2
PFS rate	0.69	0.54	0.88
Mean patient age	40.3 ± 14.7	53.8 ± 17.3	46.2 ± 14.1
Patient sex (male/female)	10 / 6	15 / 9	8 / 8
Complete resection	4	2	7
Radiotherapy	10	20	10
Chemotherapy	7	19	10
WHO grade II	11	5	13
WHO grade III	3	14	3
WHO grade IV	2	5	-

*IDH* isocitrate dehydrogenase, *n* number, *PFS rate* progression-free survival rate after 1 year, *WHO* World Health Organization

### Association of progression-free survival with mean tumor perfusion

The mean rCBV was 1.2 ± 0.6 in IDH-mutant astrocytic tumors, 2.7 ± 1.4 in IDH wild-type astrocytic tumors, and 1.9 ± 0.8 in oligodendrogliomas. It differed significantly between the astrocytic tumors (*p* < 0.001), oligodendrogliomas and IDH-mutant astrocytic tumors (*p* = 0.01), and oligodendrogliomas and IDH wild-type astrocytic tumors (*p* = 0.049). The duration of the progression-free interval correlated with the mean rCBV in IDH wild-type astrocytic tumors (*r* = 0.48, *p* = 0.04), but not in IDH-mutant astrocytic tumors (*r* = − 0.05, *p* = 0.89) and oligodendrogliomas (*r* = − 0.16, *p* = 0.63).

**Fig. 1:**
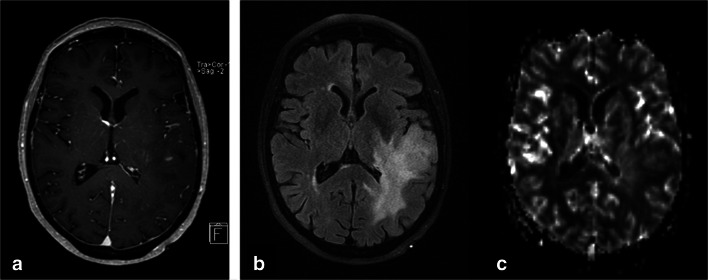
Imaging of a 74-year-old patient with IDH wild-type astrocytomas WHO grade III (FLAIR sequence, **a**) with only local contrast agent uptake (post-contrast T1-weighted sequence, **b**), and low tumor perfusion (**c**, CBV map of DSC-MRI), but tumor progression within 130 days

In predicting the 1-year PFS of patients in the group of IDH wild-type astrocytic tumors, the mean rCBV reached a sensitivity of 0.78, a specificity of 0.83, and an area under the curve (AUC) of 0.81 (cutoff value = 2.0). Patients in this group with a mean rCBV below the cutoff value (*n* = 9) had a significantly shorter 1-year PFS than those with a mean rCBV above the cutoff value (*n* = 12, *p* = 0.02). A low mean rCBV was associated with a significantly higher risk for progression (hazard ratio = 5.6; see Fig. 1 for an example).

The mean rCBV did not differ between IDH wild-type astrocytic tumors with and without MGMT methylation (*p* = 0.44), and the PFS did not differ according to the MGMT methylation status. The percentage of patients receiving adjuvant therapy was comparable in cases of IDH wild-type astrocytic tumors with high and low perfusion. Only one patient with a highly perfused wild-type astrocytic tumor and two patients with low-perfused wild-type astrocytic tumors did not undergo adjuvant treatment. The WHO grades of IDH wild-type astrocytic tumors with low and high perfusion showed similar distributions (WHO grade II *n* = 2 and *n* = 2, WHO grade III *n* = 5 and *n* = 7, WHO grade IV *n* = 2 and *n* = 3, respectively).

**Fig. 2:**
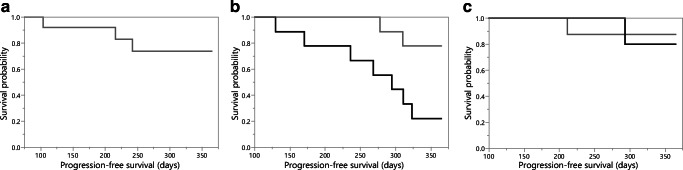
Progression-free survival of patients with mean tumor perfusion above (gray) and below (black) the cutoff value in IDH-mutant astrocytic tumors (**a**), IDH wild-type astrocytic tumors (**b**), and oligodendrogliomas (**c**). In IDH-mutant astrocytic tumors (**a**), the best cutoff value was below all measured perfusion values and limited the separation into two groups

In oligodendrogliomas, the mean rCBV could predict the 1-year PFS with a sensitivity of 1.0, a specificity of 0.43, and an AUC of 0.64 (cutoff value = 1.7). The 1-year PFS did not differ significantly in this glioma group, depending on the level of the mean rCBV (*p* = 0.87). In IDH-mutant astrocytic tumors, ROC analysis with the mean rCBV did not yield a suitable differentiation of prognostic groups as the AUC was highest when all rCBV values were above any cutoff value. Figure 2 and Table 2 show the details of the survival analyses.

**Table 2:** Survival analysis for patients with mean and maximal tumor perfusion below and above the cutoff value

Parameter	IDH-mutant astrocytoma	IDH wild-type astrocytoma	Oligodendroglioma
Mean tumor perfusion			
Total number, </> cutoff value	14/–	9/12	7/9
PFS rate, </> cutoff value	0.79/–	0.22/0.83	0.86/0.89
*p* value	–	0.02	0.87
Hazard ratio (95% CI)	–	5.6 (1.3–37.9)	1.3 (0–32.0)
Maximal tumor perfusion			
Total number, </> cutoff value	11/3	3/18	8/8
PFS rate, </> cutoff value	0.82/0.67	0/0.67	0.88/0.88
*p* value	0.63	0.01	0.87
Hazard ratio (95% CI)	0.6 (0.1–12.0)	5.1 (1.1–19.7)	10.8 (0–20.0)

*IDH* isocitrate dehydrogenase, *PFS rate* progression-free survival rate after 1 year, *CI* confidence interval

### Association of progression-free survival with maximal tumor perfusion

The maximal rCBV was 5.2 ± 3.3 in IDH-mutant astrocytic tumors, 7.5 ± 3.1 in IDH wild-type astrocytic tumors, and 5.0 ± 1.7 in oligodendrogliomas. The maximal rCBV differed significantly between with IDH wild-type and IDH-mutant astrocytic tumors (*p* < 0.003) and between oligodendrogliomas and IDH wild-type astrocytic tumors (*p* = 0.005). However, there was no significant difference between oligodendrogliomas and IDH-mutant astrocytic tumors (*p* = 0.48). The duration of the progression-free interval did not correlate with the maximal rCBV in any glioma group (IDH-mutant astrocytic tumors *r* = − 0.8, *p* = 0.83, IDH wild-type astrocytic tumors *r* = 0.14, *p* = 0.59, oligodendrogliomas *r* = − 0.18, *p* = 0.59, respectively).

In the IDH wild-type astrocytic tumor group, the maximal rCBV reached a sensitivity of 0.33, specificity of 1.0, and AUC of 0.61 in predicting the 1-year PFS (cutoff value = 4.4). Patients with a maximal rCBV below the cutoff value within these tumor group had a significantly shorter 1-year PFS (*p* = 0.01) and a higher risk for progression (hazard ratio 5.1, *p* = 0.04) than patients with a maximal rCBV above the cutoff value. The maximal rCBV did not differ between IDH wild-type astrocytic tumors with and without MGMT methylation (*p* = 0.16).

The percentage of patients receiving adjuvant therapy was comparable in wild-type astrocytic tumors with high and low perfusion. 15 of 18 patients with high maximal rCBV and all patients with low maximal rCBV underwent adjuvant treatment. The WHO grades in IDH wild-type astrocytic tumors with low and high maximal rCBV showed a similar distribution. WHO grade III was dominant in both groups.

**Fig. 3:**
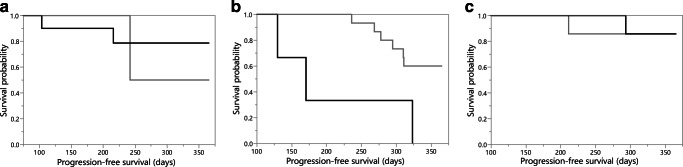
Progression-free survival of patients with maximal tumor perfusion above (gray) and below (black) the cutoff value in IDH-mutant astrocytic tumors (**a**), IDH wild-type astrocytic tumors (**b**), and oligodendrogliomas (**c**)

In oligodendrogliomas, the maximal rCBV predicted the 1-year PFS with a sensitivity of 1.0, specificity of 0.43, and AUC of 0.55 (cutoff value = 4.8). In IDH-mutant astrocytic tumors, the maximal rCBV reached a sensitivity of 0.67, specificity of 0.81, and AUC of 0.60 (cutoff value = 5.6). The 1-year PFS within the oligodendroglioma and IDH-mutant astrocytic tumor groups did not differ significantly depending on the level of the maximal rCBV (*p* = 0.87 and *p* = 0.63, respectively). Figure 3 and Table 2 show the details of the survival analysis.

## Discussion

The integrated 2016 CNS WHO of gliomas considers molecular tumor characteristics as critical important prognostic factors beyond histological WHO grades [1, 22, 23]. According to previous reports, patients with oligodendrogliomas tended to have longer PFS than patients with IDH-mutant astrocytic tumors in our cohort, and IDH wild-type astrocytic tumors had the most unfavorable prognosis [24, 25]. The potential confounders age, sex, and the extent of surgical resection were not associated with PFS in the present study [10, 11, 26]. IDH wild-type astrocytic tumors displayed the highest perfusion, followed by oligodendrogliomas and IDH-mutant astrocytic tumors. This confirms previous reports that perfusion is a marker for malignancy and the molecular characteristics of diffuse gliomas [5, 13, 25, 27].

However, DSC-MRI may be dispensable for the identification of the molecular tumor characteristics because they are assessed by pathological examination in daily routine. Therefore, we investigated the potential of DSC-MRI in predicting the PFS as an independent factor within the single molecular glioma groups. Knowledge about DSC-MRI in this context is limited, and previous studies concentrated on the old WHO classification 2007 or the subgroup of WHO grade IV glioblastomas [9, 11]. Generally, lower tumor perfusion is related to longer survival [7, 8, 28].

In oligodendrogliomas, the extent of tumor perfusion did not predict the short-term prognosis. This finding has been previously reported and might be due to the overall high perfusion and homogeneity of oligodendrogliomas [7, 10, 24]. Likewise, the extent of tumor perfusion did not have any impact on the progression-free survival within the group of IDH-mutant astrocytic tumors. The favorable prognosis and thus the low progression rate of both glioma groups may hamper our results [24]. Future studies on the long-term prognosis might give new insights.

In IDH wild-type astrocytic tumors, the extent of tumor perfusion had an impact on the short-term prognosis. Interestingly, low perfusion was associated with a three to fourfold lower 1-year PFS rate than higher perfusion and significantly shorter survival. The main proportion in this group was WHO grade III astrocytic tumors and not typical WHO grade IV glioblastomas, so our results might apply to them in particular.

The MGMT methylation status, which is an important prognostic factor in glioblastomas and might be a potential confounder, did not differ between the low- and highly perfused IDH wild-type astrocytic tumors [29]. Furthermore, the therapy regimen was similar in both groups, which excludes relevant bias by therapy on our results. Cimino et al. recently reported that IDH wild-type glioblastomas comprise several further molecular subgroups with different prognosis [24]. These subgroups might display different vascularization patterns, as seen in the present study. Additionally, low-perfused tumors might have a worse response to adjuvant therapy due to diminished delivery of chemotherapeutic agents and higher resistance to radiotherapy in hypoxic and low-perfused areas [30]. Overall, our results suggest that higher vascularization does not always result in a worse prognosis in diffuse gliomas.

The mean perfusion values of the whole tumor volume showed better performance and higher discriminatory power than maximal perfusion values. Measuring the perfusion in a hotspot is a usual way to assess DSC-MRI in brain tumors. However, our cohort comprised low-grade tumors without contrast enhancement and occasionally lower perfusion than the healthy tissue. The hotspot method does not work in this case, and including the whole tumor volume in the measurement might represent the behavior of the tumor more thoroughly [20, 21, 31].

DSC-MRI is a widely available, robust, and fast MR perfusion technique [32]. It is the best established and investigated perfusion technique for gliomas. However, DSC-MRI is prone to bias due to contrast agent extravasation from leakage through the blood-brain barrier, and appropriate correction is needed. Additionally, its evaluation is user-dependent, and the diagnostic quality is limited in regions with susceptibility artifacts [32]. As an alternative, dynamic contrast-enhanced imaging is based on T1-weighted images and can quantify the microvascular permeability [32]. Arterial spin labeling measures the perfusion without contrast agent application and is independent of bias from leakage through the blood-brain barrier [21]. It has shown promising results in the initial diagnosis and detection of recurrent disease in gliomas [33, 34].

One limitation of the present study was the small number of patients in the different glioma groups since advanced imaging, including DSC-MRI, is not performed on all patients preoperatively in our institution. Usually, only complex cases undergo advanced MRI for biopsy planning, tumor characterization, and assessment of the tumor extent. This preselection might have biased our results. Tumor progression was not confirmed by histopathology but only by imaging according to the clinical standard. We defined tumor progress by the RANO criteria in order to avoid the inclusion of cases with pseudoprogression.

In conclusion, high tumor perfusion upon initial diagnosis is not compellingly associated with worse short-term prognosis within different molecular glioma subgroups. In the overall highly perfused IDH wild-type astrocytic tumors, there are tumors with low perfusion but unfavorable prognosis.
