# Correction to: Association of dynamic susceptibility magnetic resonance imaging at initial tumor diagnosis with the prognosis of different molecular glioma subtypes

**DOI:** 10.1007/s10072-021-05352-6

**Published:** 2021-06-15

**Authors:** Cornelia Brendle, Uwe Klose, Johann-Martin Hempel, Jens Schittenhelm, Marco Skardelly, Ghazaleh Tabatabai, Ulrike Ernemann, Benjamin Bender

**Affiliations:** 1 Diagnostic and Interventional Neuroradiology, Department of Radiology, Eberhard Karls University, Hoppe-Seyler-Straße 3, 72076 Tuebingen, Germany; 2 Neuropathology, Department of Pathology and Neuropathology, Eberhard Karls University, Calwerstr. 3, 72076 Tuebingen, Germany; 3 University Hospital for Neurosurgery, Eberhard Karls University, Hoppe-Seyler-Straße 3, 72076 Tuebingen, Germany; 4 Interdisciplinary Section of Neurooncology, Eberhard Karls University, Hoppe-Seyler-Straße 3, 72076 Tuebingen, Germany

**Correction to: Neurological Sciences (2020) 41:3625–3632**

**https://doi.org/10.1007/s10072-020-04474-7**

The article “Association of dynamic susceptibility magnetic resonance imaging at initial tumor diagnosis with the prognosis of different molecular glioma subtypes”, written by Cornelia Brendle, Uwe Klose, Johann-Martin Hempel, Jens Schittenhelm, Marco Skardelly, Ghazaleh Tabatabai, Ulrike Ernemann and Benjamin Bender, was originally published Online First without Open Access. After publication in volume 41, issue 12, page 3625–3632 the author decided to opt for Open Choice and to make the article an Open Access publication. Therefore, the copyright of the article has been changed to © The Author(s) 2020 and the article is forthwith distributed under the terms of the Creative Commons Attribution 4.0 International License, which permits use, sharing, adaptation, distribution and reproduction in any medium or format, as long as you give appropriate credit to the original author(s) and the source, provide a link to the Creative Commons licence, and indicate if changes were made. The images or other third party material in this article are included in the article’s Creative Commons licence, unless indicated otherwise in a credit line to the material. If material is not included in the article’s Creative Commons licence and your intended use is not permitted by statutory regulation or exceeds the permitted use, you will need to obtain permission directly from the copyright holder. To view a copy of this licence, visit http://creativecommons.org/licenses/by/4.0.

The original article has been corrected.

